# Oxidative Stress in Fetuses and Newborns

**DOI:** 10.3390/antiox13101157

**Published:** 2024-09-25

**Authors:** Serafina Perrone, Maria Luisa Tataranno, Virginia Beretta, Giuseppe Buonocore, Eloisa Gitto

**Affiliations:** 1 Neonatology Unit, Department of Medicine and Surgery, University of Parma, 43126 Parma, Italy; virginia.beretta@unipr.it; 2 Department of Neonatology, University Medical Center Utrecht, Utrecht University, 3508 AB Utrecht, The Netherlands; m.l.tataranno-2@umcutrecht.nl; 3 Department of Molecular and Developmental Medicine, University of Siena, 53100 Siena, Italy; giuseppe.buonocore@unisi.it; 4 Department of Human Pathology in Adult and Developmental Age “Gaetano Barresi”, University of Messina, 98125 Messina, Italy; egitto@unime.it

In recent years, significant research has uncovered new mechanisms by which molecules and substances that act as free radicals generate oxidative stress in the biological system, contributing to various forms of injury and disease.

These findings warrant closer investigation. Key sources of intracellular free radicals and reactive oxygen species include mitochondrial oxidative metabolism, nitric oxide production, phospholipid metabolism, as well as proteolytic and inflammatory pathways. Without an adequate antioxidant defense system, these reactive species damage cellular components like proteins, lipids, and nucleic acids, disrupting the cell’s redox balance [1].

The progression to irreversible cellular injury involves several complex processes.

Oxidative stress has been identified as a contributing factor to a range of pregnancy-related complications [2], with long-term health consequences for the fetus. Preterm infants are particularly susceptible to oxidative damage due to their underdeveloped antioxidant systems [3].

During hypoxia-ischemia, early cellular responses, such as membrane depolarization and elevated intracellular calcium levels, lead to the activation of nitric oxide synthase and phospholipase C. These signals are further amplified by phosphorylation cascades [4]. Understanding the enzymes and substrates involved in these pathways may offer insights into cellular recovery or demise.

Oxidative stress has been linked to many complications in premature infants, including eryptosis, necrotizing enterocolitis, bronchopulmonary dysplasia, retinopathy of prematurity, intraventricular hemorrhage, respiratory distress syndrome, and kidney disease [3].

Given the breadth of these issues, the second volume of “Oxidative Stress in Fetuses and Newborns” was curated to provide neonatologists and pediatricians with a comprehensive understanding of oxidative stress in neonatal care, which remains one of the most complex and advanced fields of pediatrics.

This volume focuses on the epidemiology of neonatal mortality and morbidity, particularly highlighting conditions that expose neonates to oxidative stress. Special emphasis is placed on brain-oriented care and the early detection of perinatal abnormalities through the use of advanced laboratory techniques and equipment. Various diseases related to oxidative stress in newborns affecting the lungs, blood, immune system, and kidneys are explored. Additionally, conditions in which hypoxia is paradoxically associated with oxidative damage—due to the release of free radicals or toxic oxygen species—are discussed.

Experts in the field have contributed with their extensive knowledge, identifying current evidence-based medicine as well as areas where ongoing research is necessary. This Issue compiles three original research articles and two reviews, providing an in-depth analysis of oxidative stress in the context of modern neonatology.

Pregnancy induces significant metabolic, vascular, and physiological changes that affect the supply of essential nutrients for fetal growth and development. Overweight and obesity are the main risk factors for gestational hypertension and, in more severe cases, pre-eclampsia. Stadler JT et al. conducted an explorative study involving the overweight/obese women of the DALI cohort to assess the functionality of high-density lipoprotein (HDL) in maternal and cord blood. The key functional parameters analyzed included HDL cholesterol efflux capacity, the activity of the vaso-protective HDL-associated enzyme paraoxonase-1 (PON1), and the levels of the anti-inflammatory HDL-associated apolipoprotein M (apoM) [5]. Maternal serum samples were collected at three time points during pregnancy: at <20 weeks, at 24–28 and 35–37 weeks of gestation, and venous cord blood was drawn immediately after birth.

Their findings revealed that lower maternal PON1 activity and apoM levels during early pregnancy were linked to a higher risk of developing gestational hypertension. This condition was also associated with impaired HDL cholesterol efflux capacity and reduced PON1 activity in cord blood, potentially affecting the offspring’s vascular health.

Many studies have shown that inflammatory disorders significantly impact HDL composition and function [6,7].

Since pregnancy itself is a state of low-grade systemic inflammation, structural changes in HDL particles have been observed, particularly an increase in HDL size and altered protein composition in pregnant women between 18 and 24 weeks of gestation [8].

One of the studies presented in this Special Issue examined the key roles of HDL, including cholesterol efflux from macrophages, PON1 activity, and the involvement of apoM in hypertension and cardiac hypertrophy via G protein-coupled S1P receptors [9]. It is hypothesized that gestational hypertension impairs HDL functionality, which could negatively affect both mothers and their offspring.

Another study in this collection, by Shankar N et al., investigated the regulatory mechanisms of inflammation in developing lungs [10] using an animal model of bronchopulmonary dysplasia [BPD]. The authors discovered that hyperoxia, a major risk factor for BPD, has a negative impact on lymphatic endothelial homeostasis, decreasing the expression of prospero homeobox 1 [Prox1] and vascular endothelial growth factor c [Vegf-c] and increasing the expression of heme oxygenase 1 [HO1] and NAD[P]H dehydrogenase [quinone]1 [NQO1] in endothelial cells, reducing their tubule formation ability. Prox1 is a master nuclear transcription factor that confers the lymphatic endothelial cells’ fates, and in its absence, the lymphatic endothelial cells fail to differentiate and proliferate [11].

Vegf-c promotes the formation of initial lymphatic vessels from embryonic veins and represents a potential target for modulating lymphangiogenesis and lymphatic function under various pathological conditions [12,13].

Heme oxygenase 1 and NQO1 are strictly related to oxidative stress following hyperoxia, representing an adaptive response to attenuate the effects of oxygen exposure. Oxidative stress may be one of the primary drivers of disrupted lymphatic endothelial cell homeostasis following hyperoxia [14,15].

In a comprehensive review, You-Lin Tain and Chien-Ning Hsu examined how environmental factors contribute to oxidative stress-related renal programming. These factors include imbalanced maternal nutrition, maternal disorders like diabetes and pre-eclampsia, and exposure to environmental toxins [16].

Research shows that adverse prenatal conditions, such as maternal high-fat diets or chemical exposure, can impair kidney development, leading to a higher risk of kidney disease later in life [17,18,19,20].

The underlying mechanisms include oxidative stress, nitric oxide deficiency, low nephron numbers, dysregulation of the renin–angiotensin system, and gut microbiota dysbiosis, among others [21,22,23,24,25]. These findings highlight the importance of optimizing the prenatal environment to minimize risks.

Oxidative stress and epigenetic gene regulation during pregnancy might be crucial common factors leading to fetal renal programming of offspring [24,25].

Promoting an optimal prenatal environment to minimize early-life risk factors may not only determine optimal fetal development but may even avert oxidative stress-mediated damage to developing kidneys. Several antioxidant substances, including vitamins and melatonin, have revealed promising data in animal models, and their efficacy needs future translation into human investigations.

Additionally, the novel method introduced by Ten-Doménech I et al. for measuring the glutathione (GSH) to glutathione disulfide (GSSG) ratio in dried blood spots (DBS), a biomarker of intracellular redox status, offers a promising approach for neonatal oxidative stress monitoring. This method enables the simultaneous determination of GSH and GSSG by ultra-performance liquid chromatography–tandem mass spectrometry (LC-MS/MS) and has been shown to preserve sample integrity, making it suitable for routine clinical use [26,27,28,29,30,31,32,33].

The authors also provided the reference ranges of GSH and GSSG for healthy term infants.

Oxidative stress is a striking aggravating factor of hypoxic-ischemic brain injury.

Perrone et al. provided a review focusing on the effects of asphyxia on the neonatal brain, including changes in cerebral blood flow, oxygen consumption, and glucose metabolism [34]. The neonatal brain is particularly vulnerable to oxidative stress-induced damage, and the authors discussed serum biomarkers that could be used for the early identification of newborns at high risk of neurological disorders. While these biomarkers are not yet standardized in clinical practice, their potential for predicting disease progression is promising and should be further investigated [35,36,37].

In conclusion, this Special Issue highlights the role of oxidative stress in the perinatal period and offers valuable insights into the pathophysiology of related diseases. By presenting relevant research and recommendations, this volume aims to equip neonatologists with practical solutions for managing oxidative stress in neonates.
